# Reversal of *MYB*-dependent suppression of *MAFB* expression overrides leukaemia phenotype in MLL-rearranged AML

**DOI:** 10.1038/s41419-023-06276-z

**Published:** 2023-11-23

**Authors:** A. Negri, C. Ward, A. Bucci, G. D’Angelo, P. Cauchy, A. Radesco, A. B. Ventura, D. S. Walton, M. Clarke, B. Mandriani, S. A. Pappagallo, P. Mondelli, K. Liao, G. Gargano, G. M. Zaccaria, L. Viggiano, F. M. Lasorsa, A. Ahmed, D. Di Molfetta, G. Fiermonte, M. Cives, A. Guarini, M. C. Vegliante, S. Ciavarella, J. Frampton, G. Volpe

**Affiliations:** 1 Hematology and Cell Therapy Unit, IRCCS Istituto Tumori “Giovanni Paolo II”, Bari, Italy; 2 Edge Impulse Inc., San Jose, CA USA; 3 Max Planck Institute of Immunobiology and Epigenetics, 79108 Freiburg, Germany; 4 Clent Life Sciences, DY84HD Stourbridge, UK; 5 Institute of Cancer and Genomic Sciences, College of Medical and Dental Sciences, University of Birmingham, B152TT Birmingham, UK; 6 Department of Bioscience, Biotechnology and Environment, University of Bari “Aldo Moro”, 70125 Bari, Italy; 7 School of Biology and Biological Engineering, South China University of Technology, Guangzhou, 510006 China; 8 Department of Mathematics, University of Bari “Aldo Moro”, Bari, Italy; 9 Department of Electrical and Information Engineering, Polytechnic University of Bari, Bari, Italy; 10 Department of Biology, University of Bari “Aldo Moro”, Bari, Italy; 11 Department of Interdisciplinary Medicine, University of Bari “Aldo Moro”, Bari, Italy

**Keywords:** Acute myeloid leukaemia, Mechanisms of disease

## Abstract

The transcription factor MYB plays a pivotal role in haematopoietic homoeostasis and its aberrant expression is involved in the genesis and maintenance of acute myeloid leukaemia (AML). We have previously demonstrated that not all AML subtypes display the same dependency on *MYB* expression and that such variability is dictated by the nature of the driver mutation. However, whether this difference in MYB dependency is a general trend in AML remains to be further elucidated. Here, we investigate the role of *MYB* in human leukaemia by performing siRNA-mediated knock-down in cell line models of AML with different driver lesions. We show that the characteristic reduction in proliferation and the concomitant induction of myeloid differentiation that is observed in MLL-rearranged and t(8;21) leukaemias upon *MYB* suppression is not seen in AML cells with a complex karyotype. Transcriptome analyses revealed that MYB ablation produces consensual increase of *MAFB* expression in MYB-dependent cells and, interestingly, the ectopic expression of *MAFB* could phenocopy the effect of *MYB* suppression. Accordingly, in silico stratification analyses of molecular data from AML patients revealed a reciprocal relationship between *MYB* and *MAFB* expression, highlighting a novel biological interconnection between these two factors in AML and supporting new rationales of *MAFB* targeting in MLL-rearranged leukaemias.

## Introduction

The MYB proto-oncoprotein is a sequence specific transcription factor that is highly expressed in immature haematopoietic progenitors and exerts master regulator functions during normal blood development. It controls the activation of genes that are necessary for definitive haematopoiesis, maintenance of stem cell self-renewal, and myeloid lineage specification and differentiation [1–5].

Transcriptional dysregulation of MYB has been shown to be a central event in perpetuating a malignant self-renewal programme coupled with an arrest in normal myeloid differentiation, which are necessary events in the initiation and the progression of different types of leukaemia. The involvement of MYB in the development of myeloid diseases was originally demonstrated through the capacity of retrovirus-derived sequences to transform haematopoietic cells [6, 7]. This was followed by the identification of duplication of the *MYB* locus in paediatric acute lymphoblastic leukaemia [8, 9] and of genomic rearrangements involving the *MYB* gene in acute basophilic and myelomonocytic leukaemia [10–12]. Moreover, *MYB* has been shown to be an essential downstream target of multiple known oncogenic proteins such as HOXA9 and its TALE partners MEIS1 and PBX1 [13, 14]. These latter factors are themselves downstream targets of MLL fusion proteins [13, 14] and *Myb* was demonstrated to be a strong mediator of oncogenic addiction in both cell lines and mouse models of MLL-AF9 driven leukaemia [15]. Previous studies from our group demonstrated that one mechanism through which MYB influences AML establishment involves the enforcement of sustained expression of *FLT3*, which is mediated through a functional cooperation with C/EBPα [4, 16]. Furthermore, we have also demonstrated that the dependency on *Myb* generally observed in leukaemia is attenuated in a murine model of AML that is driven by biallelic CEBPA N-terminal mutations [17].

Here, we sought to assess in more detail how the leukaemia phenotype-related dependence on *MYB* translates to human leukaemia that are driven by different genetic lesions. In this pursuit, we focussed on human AML driven by MLL-rearrangements, RUNX1-ETO translocations and those characterized by complex karyotypic lesions and assessed if and how those different leukaemia contexts would display a characteristic phenotypic modulation in response to *MYB* suppression. In agreement with previous reports [15], we show that depletion of *MYB* reverses the leukaemia-associated cellular phenotypes in MLL-rearranged AML, which is accompanied by de-repression of *MAFB*. However, we find that AML with complex karyotypic lesions does not undergo the conventional transcriptional and morphological alterations that are associated with *MYB* suppression. Importantly, we demonstrated that ectopic expression of *MAFB* in a panel of AML cells lines leads to an even stronger phenotypic modulation compared to *MYB* ablation. We analysed gene expression profiling data from cohorts of patients with either MLL-rearrangements or complex karyotypes categorising those based on the expression of either *MYB* or *MAFB*. In line with data obtained in cell lines, analysis of gene expression profiles showed that patients with low *MYB* expression and high *MAFB* levels display a substantial overlap in their expression signature.

## Material and methods

### Cell lines

KASUMI1, and MOLM14 were purchased from Deutsche Sammlung von Mikroorganismen und Zellkulturen. KG1A, MV4-11 and THP1 were obtained from the American Type Culture Collection. FUJIOKA cells were purchased from Japanese Collection of Research Bioresources Cell Bank. FUJIOKA, KASUMI1, MOLM14 and THP1 cells were cultured in RPMI-1640 medium supplemented with 10% foetal bovine serum (FBS), 50U/ml penicillin, 50 µg/ml streptomycin, 2mM L-glutamine. THP1 were further supplemented with 0.05 mM of 2-mercaptoethanol (Sigma Aldrich). KG1A cells were cultured in RPMI-1640 supplemented with 20% FBS, while MV4-11 cells were grown in IMDM medium. Cells were maintained at 0.5 × 10^6^ cells/ml at 37 °C with 5% CO_2_ in a humidified incubator and were washed with phosphate buffered saline solution between passages. All cell lines have been routinely tested for mycoplasma.

### Transfection experiments, proliferation, and differentiation assays

In total, 5 × 10^6^ FUJIOKA, KG1A, KASUMI1, THP1, MV4-11 or MOLM14 cells were electroporated using an EPI 3500 (Fischer, Germany) single 250 V pulse for 10 ms with 300 mM of *MYB* siRNA or a scrambled negative control siRNA (SIGMA ALDRICH) as previously reported in Clarke et al. [5]. After electroporation, cells were kept in the electroporation cuvette for 10 min after which cells were added RPMI-1640 with 10% or 20% FBS, supplemented with penicillin/streptomycin and L-glutamine at a concentration of 10^6^ cells per ml and returned to an incubator kept at 37 °C and 5% CO_2_. For the MAFB ectopic expression, its coding sequence was amplified by PCR using the MAFB (NM_005461) human untagged clone (OriGene, SC116756) as template. The forward (5ʹ-CCTAGGGCCACCATGGCCGCGGAGCTG-3ʹ) and reverse (5ʹ- GGATCCTTACAGAAAGAACTCGGGAGAGGAG-3ʹ) oligos carried an AvrII and BamHI restriction sites, respectively, which allowed the cloning of the amplified fragment in the lentiviral expression vector pCW57-GFP-2A-MCS (Addgene, plasmid no. 71783), digested with NheI and BamHI, under the control of the doxycycline inducible Tet-responsive promoter PTight [18]. The resulting plasmid was sequence verified. Recombinant lentiviral particles were produced following previously published methods [19]. Cells were transfected with lentiviral particles carrying the empty vector, used as control, or the one expressing MAFB and selected with puromycin. After transfection (MYB silencing) or after dox induction (MAFB ectopic expression) cells were plated at a density 10^6^ cells/ml and viable cells were counted and passaged at a ratio of 1:2 every 24 h for four consecutive days to determine their proliferative capacity. Assessment of differentiation at 96 h post *MYB* knock-down or *MAFB* induction was achieved by flow cytometry staining of the cells with anti-CD11b PE-Cy7 and CD14 APC (eBioscience). Acquisition and analysis of flow cytometric data was performed using Cyan ADP with Flow Jo software.

### Quantitative RT-PCR and western blot

10^6^ cells from FUJIOKA, KG1A, KASUMI1, MOLM14, MV4-11 and THP1 cells lines were collected 24 h post transfection or dox induction and RNA was extracted using RNeasy Mini kit (QIAGEN), and first-strand cDNA synthesis was performed using standard protocols. Quantitative RT-PCR analysis for *MYB* and *MAFB* was performed using predesigned Taqman gene expression assays (Applied Biosystems). Total protein lysates obtained from transfected cells were used for Western Blot analysis using the following antibodies: anti-MYB mouse monoclonal (1:1000, Upstate/Millipore) and anti-GAPDH mouse monoclonal (1:10000 dilution, Abcam). Western blot quantification was performed using Image J.

### Statistical analysis

**Fig. 1:**
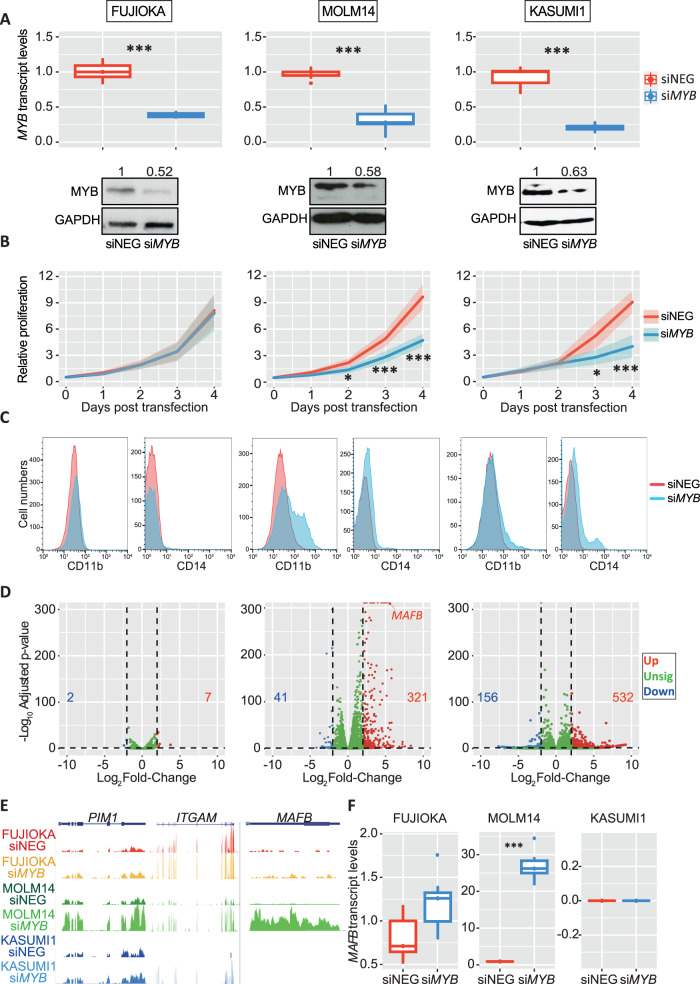
*MYB* depletion in AML driven by MLL-rearrangements reverses their leukaemia phenotype. **A** *MYB* knock-down efficiency in FUJIOKA, MOLM14 and KASUMI1 cells was determined 24 h post transfection by quantitative-PCR and immunoblotting in cells transfected with *MYB* siRNA or scrambled negative control. **B** Line plot representing cell viability determined by counting cells every 24 h for 4 consecutive days. Results are representative of 3 independent experiments. The shaded lines incorporate the standard deviation. Statistical analysis was calculated using Student’s *t* test. (***p < 0.001 *p < 0.05). **C** Histograms representing the analysis of CD11b and CD14 myeloid surface marker expression in cell lines transfected with either *MYB* siRNA or corresponding scrambled negative control. **D** Volcano plot representing the differentially regulated genes in the response to *MYB* siRNA treatment. Up-regulated (over 2 Log_2_ FC) and down-regulated (below -2 Log_2_ FC) are indicated by red and blue dots, respectively, and the total number of genes is indicated in each plot. **E** UCSC genome browser screenshots of RNA-seq performed in FUJIOKA, MOLM14 and KASUMI1 cells with either siNEG or si*MYB* treatment at *PIM1*, *ITGAM* and *MAFB* loci. Profiles are scaled to 1% GAPDH. **F** qPCR analysis of *MAFB* transcript abundance in all cell lines transfected with either siNEG or si*MYB*. This analysis represents an average of 3 independent experiments. The statistical significance shown in the plot was calculated using Student’s *t* test. (***p < 0.001).

Statistical significance was performed by applying Student’s *t* test for pairwise comparison and the *p* values are indicated where appropriate. Analysis of *MYB* and *MAFB* expression in human patient microarray data from the Haferlach cohort presented in Figs. 1A, 4A was performed using non-parametric Kruskal-Wallis test. All statistical analysis was performed using Graphpad Prism 7 (Graphpad Software Inc).

### RNA-sequencing

For RNA-Seq, libraries were prepared using the Illumina TruSeq Stranded kit according to the manufacturer’s instructions. Sequencing was performed in Genomics facility of the Institute of Cancer and Genomic Sciences, University of Birmingham, Birmingham, UK on an Illumina NextSeq 500 instrument.

### RNA-seq and differential gene expression analysis

Reads were trimmed using Trim Galore v 0.5.0 with --paired option. Gene counts were calculated using RSEM v1.2.22 using rsem-calculate-expression with --bowtie2, --bowtie2-sensitivity-level very sensitive, --output-genome-bam and --paired-end options. The reads were aligned to hg38 assembly indexed with version 81 Ensembl GTF. Counts were normalized using DESeq2 v1.24 in R v3.6.0. Differential expression analysis was performed using the deseq function in DESeq2. Genes were considered significant if their adjusted p.value was less than 0.05 and their Log2 fold change was greater than 2 or less than -2. Heatmaps were generated using the pheatmap v1.0.12 package and the pheatmap function. Euler diagrams were produced using the VennDiagram v1.6.20 package and the venn.diagram function using the intersected significantly up-regulated genes or the significantly down-regulated genes. Volcano plots were produced using the ggplot2 v3.2.1 package.

### Genome browser tracks

The genomic bam output from RSEM was used in the deeptools v3.1.3 bamCoverage function with --normalizeUsing CPM and the -bl hg38.blacklist.bed options. The resulting bigwig file was uploaded to a web server and visualised on UCSC genome browser.

### Patient profiling array and data processing

GSE13204 [20] normalized data was downloaded from NCBI Gene Expression Omnibus (GEO) while the data from the TARGET-AML cohort were retrieved from Genomic Data Commons under the dbGaP study accession number phs000465.v18.p7 [21]. Patients were ranked for high or low *MYB* or *MAFB* expression using quantile cut off values as follows: 75^th^ percentile and higher were considered high expressers while 25th percentile and lower were considered low expressers. Patients were subclassified and gene differential gene expression analysis was carried out as previously reported [22, 23].

## Results

### Reduction of *MYB* expression allows MLL-rearranged leukaemia cells to differentiate but does not reverse the block in complex karyotype AML cells

We have previously demonstrated the requirement for *Myb* in the maintenance of murine CEBPA-driven leukaemia and that the dependency on *Myb* expression is dictated by the nature of the mutations that drive the leukaemic phenotype [16, 17]. To assess the relevance of our previous findings to human leukaemia and to test whether different leukaemia subtypes show a different dependency of *MYB* expression as observed in the case of CEBPA mutations, we analysed publicly available array data [20] by classifying cases based on their karyotypic abnormalities. In line with previous reports, we found the highest *MYB* expression in AML characterized by MLL fusions and RUNX1-ETO translocation as compared with healthy bone marrow donors, whereas no differences were observed in patients with Inv [16] lesions and abnormal/complex karyotype (Figure S1A). As such, we sought to assess how reducing *MYB* levels would impact on the maintenance of leukaemia types that are characterized by different levels of *MYB* expression, focusing on leukaemias driven either by MLL fusions, t(8;21) translocations or complex karyotypes. We employed MOLM14, MV4-11 and THP1 representing well characterized models of MLL-driven leukaemia, KASUMI1 as a widely used line for RUNX1-ETO fusion, and FUJIOKA and KG1A cells as models for complex karyotype leukaemias.

To determine the immediate consequences of *MYB* reduction in the two classes of AML, we transfected the cell lines with siRNA targeting either *MYB* or a scrambled negative control. Efficient reduction in MYB expression was verified at 24 h post transfection, resulting in a 65–80% knockdown in all cell lines (Fig. 1A). Transfected cells were cultured for up to 96 h, determining cell number daily to assess whether *MYB* reduction affects proliferation capacity. Decreased *MYB* levels led to a pronounced growth retardation in MOLM14, THP1, MV4-11 and KASUMI1, confirming the dependency of these cells on MYB expression [15]. Conversely, no proliferation defect was observed neither in FUJIOKA nor in KG1A cells (Fig. 1B, S1B). As we have previously demonstrated that MYB manipulation in different leukaemia phenotypes can override the differentiation block to instruct a myeloid commitment programme, we performed flow cytometric analysis to test the cell differentiation capacity by measuring CD11b and CD14 surface markers, observing a shift in the expression of myeloid markers in MOLM14 and KASUMI1, but not in FUJIOKA cells, suggesting a lower extent of *MYB* expression dependency in leukaemias with complex karyotype (Fig. 1C).

### Molecular consequences of *MYB* reduction in MLL-driven and complex karyotype AML

Previous studies have reported the capacity of *MYB* to influence leukaemia establishment and maintenance through enforcing a self-renewal program while suppressing myeloid commitment [15, 17, 24, 25]. To better understand the process of how *MYB* reduction could lead to such a different response in different leukaemia settings, we explored the transcriptomes of FUJIOKA, MOLM14 and KASUMI1 cells 24 h after *MYB* silencing. For this analysis, we considered up-regulated genes as those displaying an average Log_2_ fold change (FC) above 2 and the down-regulated ones with values below -2, with an adjusted *p* value < 0.05. We observed that *MYB* knock-down did not induce any broad changes in gene expression of FUJIOKA cells, with only 7 genes being up-regulated and 2 genes being down-regulated. In contrast, we observed in MOLM14 and KASUMI1 41 and 156 down-regulated and 321 and 532 up-regulated genes, respectively (Fig. 1D). These finding are in agreement with previous reports of *MYB*-induced gene repression in myeloid cells. *MYB* knock-down in MOLM14 cells recapitulated the typical pattern of upregulation (*PIM1*, *ITGAM/CD11b*, *CD14*, *S100A9* and *DUSP6*) and downregulation (*MYC*, *BCL2*, *CCND2* and *GFI1*) of myeloid genes that are known targets of *MYB* [25, 26] (Fig. 1E, S2A). Among others, we observed a striking increase in the expression of *MAFB*, which is a transcription factor primarily involved in myeloid differentiation and in several haematological malignancies [27–29]. The up-regulation of *MAFB* in response to *MYB* reduction was also confirmed by quantitative PCR analysis in all cell lines carrying MLL rearrangements, while no significant difference was observed in complex karyotype cells. qPCR analysis failed to detect any *MAFB* mRNA expression in KASUMI1 cells (Fig. 1F, S1B). This latter finding, together with the observation of only a small overlap between the differentially expressed genes in MOLM14 and KASUMI1 (Fig. S2B), is intriguing as it suggests that these cells could respond to *MYB* suppression through different mechanisms. These observations are in line with previous work in which a boost in *MAFB* expression was observed upon pharmacological inhibition of MYB in MLL-r cell lines [30, 31] (Fig. S2C). Furthermore, we also inspected ChIP-seq data generated from MV4-11 cells that have undergone MYB peptidomimetics interference, showing a significant depletion of MYB binding from the MAFB locus (Fig. S2D).

Our data prompt the idea that up-regulation of *MAFB* could be a potential mechanism by which *MYB* reduction affects the malignant cell phenotype in MLL-rearranged leukaemia.

### *MAFB* ectopic expression phenocopies the effects of *MYB* suppression in MLL-mutant AML cell lines

Given the striking upregulation of *MAFB* in response to *MYB* suppression, we reasoned that ectopic expression of *MAFB* could phenocopy the consequences of MYB inhibition and, as such, could represent a novel therapeutic avenue for the treatment of MLL-rearranged leukaemias. In this pursuit, we transduced our AML cell lines with a construct harbouring the full *MAFB* cDNA, or the corresponding empty vector, under the control of a Tet responsive element to generate Dox-inducible cell lines. To have more robust observations, we also used another MLL-rearranged cell line, namely THP1, in which the dependency of this class of leukaemia has been largely investigated [15, 32, 33].

**Fig. 2:**
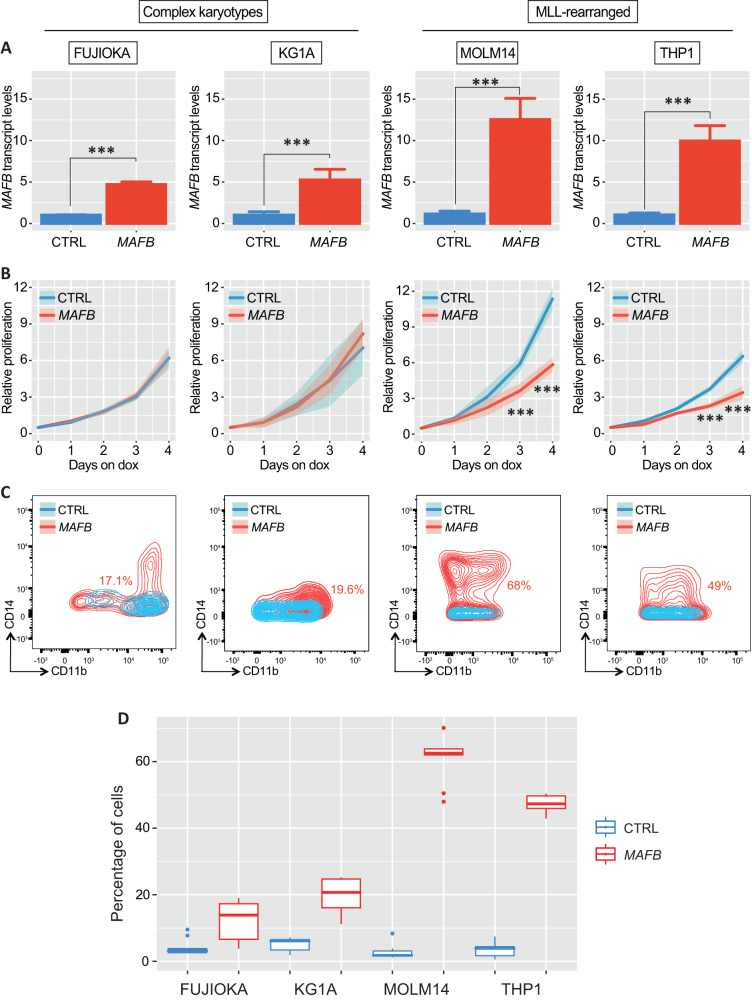
Phenotypic consequences of *MAFB* ectopic expression in human AML cell lines. **A** Bar plot showing *MAFB* transcript qPCR quantification in complex karyotype (left) and MLL-rearranged (right) cell lines 48 h post doxycycline induction. **B** Line plot representing cell viability determined by counting cells every day for 96 h on constitutive doxycycline administration. The shaded lines incorporate the standard deviation. Statistical analysis was calculated using Student’s *t* test. (***p < 0.001). **C** Two-dimensional dot plot showing the analysis of CD11b and CD14 myeloid surface marker expression in all cell lines 96 h after ectopic MAFB expression. The percentage of double positive cells is indicated in each plot. Results are representative of 3 independent experiments. **D** Boxplot representing the percentage of myeloid cells generated upon MAFB overexpression in all cell lines. Control cells are indicated in blue while the red boxplot represent cells lines in which MAFB was ectopically expressed.

The successful ectopic expression of *MAFB* was verified by qPCR at 48 h post Dox induction, displaying roughly a 5-fold higher expression relative to the control in complex karyotype cells lines and being 10-fold higher in MLL-rearranged cell lines (Fig. 2A). We also tried to generate Dox-inducible KASUMI1 cells, however, despite using the same system, we still failed to detect any *MAFB* expression upon dox stimulation (data not shown).

Given that MYB deficient MLL-mutant cell lines displayed a marked growth retardation, we proceeded to evaluate their proliferative capacity post *MAFB* induction by counting those cells daily. We noticed that, while no difference in terms of growth was observed neither in FUJIOKA nor in KG1A cell lines, both MOLM14 and THP1 also displayed a substantial proliferative defect (Fig. 2B).

Similarly, the measurement of myeloid maturation markers CD11b and CD14 at 96 h post Dox induction highlighted a remarkable boost in myeloid commitment in both MOLM14 and THP1 cells, in particular for the expression of CD14, this being between 50% and 60% higher than that of the control. Conversely, little evidence of myeloid maturation was observed in KG1A and FUJIOKA cell lines, respectively (Fig. 2C). This phenomenon was observed in a number of replicates and is depicted in the boxplot in panel 2D.

These findings suggest that the elevation of *MAFB* levels may mimic *MYB* ablation in MLL-mutant leukaemias.

### High *MAFB* expression phenocopies the transcriptional consequences of lowering *MYB* expression in human AML patients carrying MLL mutations

**Fig. 3:**
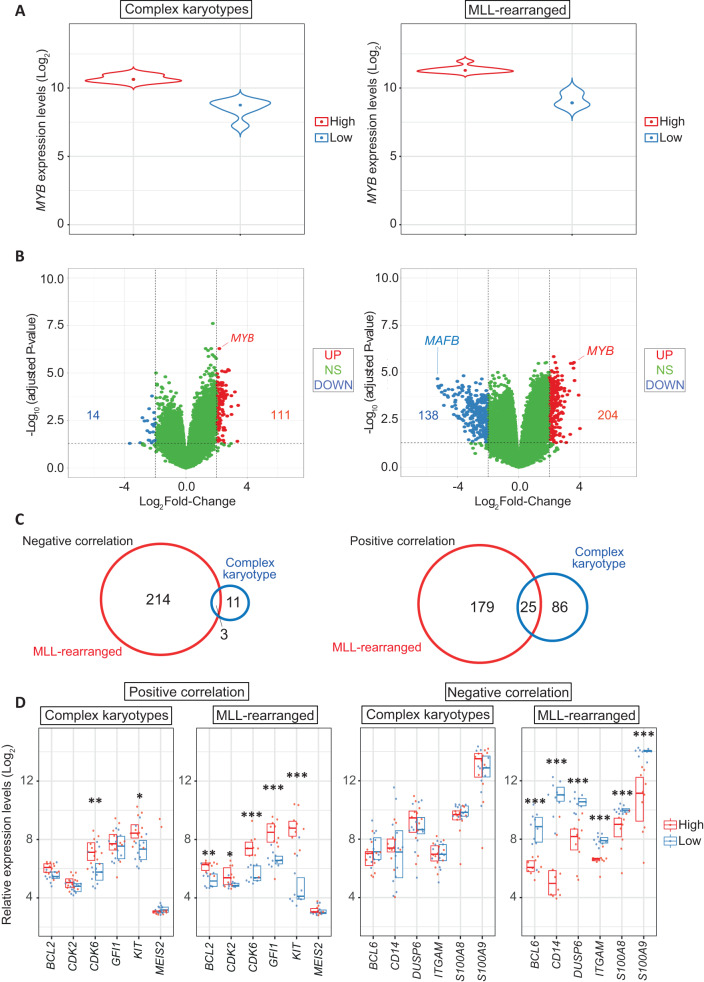
Validation of siRNA-mediated *MYB* knock-down consequences in AML patient expression arrays. **A** Violin plot representing the boundaries of *MYB*^low^ (lower quartile, 0–25% of expression range) and *MYB*^high^ (upper quartile, 75–100% of expression range) patients in the subgroups with complex karyotype (left panel) or MLL-rearrangements (right panel) from the cohort reported by Haferlach et al. **B** Volcano plot representing differentially expressed genes that display either negative (below -2 Log_2_ FC, indicated as blue dots) or positive (above 2 Log_2_ FC, indicated as red dots) correlation when comparing high versus low expressers in each AML subgroup. **C** Euler diagrams showing the numbers and overlap of genes that are either negatively (left panel) or positively (right panel) correlated with *MYB* expression when comparing complex karyotype and MLL-rearrangements. **D** Expression of representative *MYB* myeloid target genes (*BCL2, CDK2, CDK6, GFI1, KIT, MEIS2, BCL6, CD14, DUSP6, ITGAM, S100A8*, and *S100A9*) in both subgroups. Data are presented as overlapping scatter plots in which *MYB*^high^ patients are indicated in red and *MYB*^low^ patients are indicated in blue. Every plot shows a colour-coded boxplot showing a median interquartile range. The statistical analysis shown in each plot indicates *p* value adjusted for false discovery rates (***< 0.001, **< 0.01, *< 0.05).

We next proceeded to validate our findings on subgroups of patients characterized by either abnormal karyotypes or MLL-rearrangements from the dataset by Haferlach et al. [20]. Patients were ranked within each subgroup according to *MYB* expression and selected the bottom and the top quartiles of the whole expression range as low and high expressers, respectively (Fig. 3A). We performed differential gene expression analysis and identified 204 versus 111 genes that displayed a positive correlation with *MYB* expression and 217 versus 14 genes that displayed a negative correlation in the subgroups characterized by MLL aberrations and complex karyotype, respectively (Fig. 3B). In line with our experimental observations, this correlative analysis suggested that MLL patients display a greater dependency on *MYB* expression, while only minor transcriptome differences were observed among complex karyotype patients. Importantly, we observed *MAFB* to be among the most negatively correlated of genes (-5.7 FC), thus strengthening the hypothesis that MAFB could mediate the strong influence of *MYB* depletion on leukaemia progression. Intersection of transcriptome differences within the subgroups of patients showed only a minimal overlap, this being 1.38% versus 21% and 12% versus 22% for the negatively and positively correlated genes (Fig. 3C), respectively, further suggesting that *MYB* reduction in different subgroups can lead to quite different outcomes. To investigate this in more detail, we screened the subgroups of patients by looking at the expression of typical myeloid genes that are known MYB targets; we found marked differences in the expression of several genes that are normally either positively (e.g. *BCL2, CDK6*, *FLT3, GFI1* and *KIT* and *MEIS2*) or negatively regulated (such as *BCL6*, *CD14*, *DUSP6, ITGAM, S100A8* and *S100A9*) by MYB, in MLL-rearranged leukaemia patients, while only minimal differences were observed for the same genes in the complex karyotype subgroup when comparing *MYB*-high versus *MYB*-low subgroups (Fig. 3D).

**Fig. 4:**
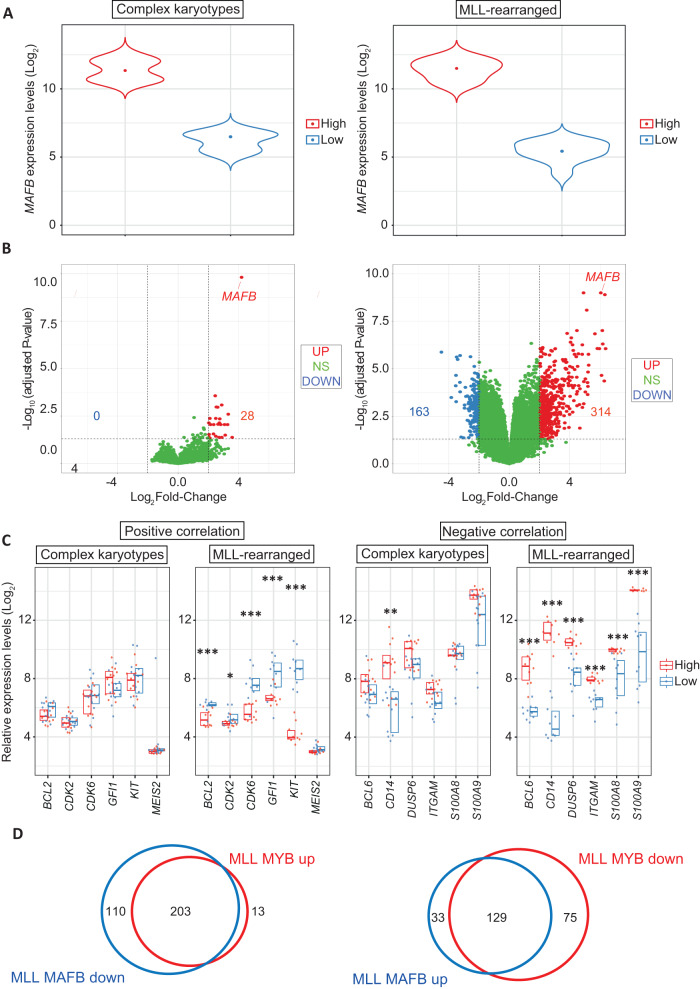
Correlation of the molecular consequences of high *MAFB* and low *MYB* in AML patients with MLL-rearrangements. **A** Violin plot representing the boundaries of *MAFB*^low^ (lower quartile, 0–25% of expression range) and *MAFB*^high^ (upper quantile, 75–100% of expression range) patients in the subgroups with complex karyotype (left) and MLL-rearrangements (right) from the Haferlach cohort. **B** Volcano plot indicating differentially expressed mRNAs with either negative (below -2 Log_2_ FC, indicated as blue dots) or positive (above 2 Log_2_ FC, indicated as red dots) correlation when comparing high versus low *MAFB* expressers in each AML subgroup. **C** Differential expression of representative *MYB* myeloid target genes (*BCL2, CDK2, CDK6, GFI1, KIT, MEIS2, BCL6, CD14, DUSP6, ITGAM, S100A8*, and *S100A9*) comparing low versus high expressers in both complex karyotype and MLL-rearrangements subgroups. Data are presented as overlapping scatter plots in which *MAFB*^high^ patients are indicated in red and *MAFB*^low^ patients are indicated in blue. Every plot shows a colour-coded boxplot showing a median interquartile range. The statistical analysis shown in each plot indicates p-value adjusted for false discovery rates (***< 0.001, **< 0.01, *< 0.05). **D** Euler diagrams indicating the numbers and overlap of genes that display negative correlation with *MAFB* and positive correlation with *MYB* (left panel) or genes that display positive correlation with *MAFB* and negative correlation with *MYB* (right panel) in the subgroup of patients carrying MLL-rearrangements.

To compare the consequences of different *MAFB* levels, we selected high and low *MAFB* expressers patients from the MLL and complex karyotype subgroups (Fig. 4A) and performed differential gene expression analysis using the same parameters described for the *MYB* classification. We observed a large number of gene transcript differences in the patients of the MLL cohort (314 positively and 163 negatively correlated genes) while only a small set of differences was observed in complex karyotype patients (28 positively and no negatively correlated genes) (Fig. 4B).

We looked at the link between high *MAFB* expression and known target genes of *MYB* in the context of myeloid cells. This revealed that in MLL-rearranged patients, genes that are positively correlated with *MYB* are generally negatively correlated with *MAFB*, such as *BCL6*, *CD14, S100A8* and *S100A9*. Conversely, *CDK6*, *FLT3*, *GFI1*, *KIT*, and *MEIS2* that are negatively correlated with *MYB* show a positive correlation with *MAFB* (Fig. 4C). Furthermore, we observed a generally large overlap between the genes positively correlated with *MYB* and negatively correlated with *MAFB* and between genes negatively correlated with by *MYB* and positively correlated with *MAFB* (Fig. 4D).

To strengthen our observations, we retrieved RNA-seq data from the TARGET-AML cohort and performed that same stratification on complex karyotype or MLL-rearranged patients based on either *MYB* or *MAFB* expression. In keeping, we noticed that MLL-rearranged patients displayed a marked anti-correlation between *MYB* and *MAFB* (Fig. S4A) as well as the typical expression changes for canonical MYB target genes (Fig. S4B). Similar to the analysis from the Haferlach cohort, we observed that genes positively correlated with MYB displayed a negative correlation with MAFB and vice versa (Fig. S4C, D). In contrast, the same analysis performed in patients with complex karyotypes revealed a larger number of differentially regulated genes, both when dichotomising patients based of *MYB* and *MAFB* expression, although no significant association between those two genes was observed (Fig. S4B, D).

Our data highlight a reciprocal correlation between the expression of *MYB* and *MAFB* in MLL-rearranged AML, thus prompting further studies on *MAFB* as useful molecular predictor toward *MYB* pharmacological depletion or inhibition.

## Discussion

We have previously shown that manipulating *Myb* expression levels in murine leukaemia driven by biallelic CEBPA mutations have profound effects on both the self-renewal and differentiation block of leukaemia stem cells and that the dependency on Myb expression is dictated by the nature of the mutations that drive the disease.

In the present study, we used siRNA-mediated *MYB* silencing and transcriptome profiling to explore the oncogenic addiction to *MYB* levels of human leukaemia driven by different genetic lesions. By taking this approach in cell lines modelling the main leukaemia classes, that is MLL-rearrangements, t(8;21) translocations, and complex karyotypes, we have shown that while MLL-driven AML requires *MYB* to enforce self-renewal and a myeloid differentiation barrier, complex karyotype leukaemia shows a reduced dependency on *MYB* expression.

In line with previous observations from our work on CEBPA mutant models [17], we show a very different phenotypic response to *MYB* depletion in MOLM14 and FUJIOKA cell lines, which is reflected in distinct modulations of the transcriptome. In fact, we show that perturbation of *MYB* expression is able to reverse the differentiation block normally observed in leukaemia and impairs the self-renewal capacity of MOLM14 cells, while such a reduction in *MYB* appears to be better tolerated in FUJIOKA cells as the undifferentiated state persists and the differentiation block is not overcome. A similar phenomenon is also observed in KG1A cells, another widely used cell line modelling complex karyotype leukaemia, in which no change in proliferation or myeloid commitment dynamics is observed. Conversely, KASUMI1 cells modelling the t(8;21) phenotype, exhibit a similar response to that observed in the MOLM14 line.

A potential limitation of our study is that we used siRNAs that have led to a substantial transcript downregulation although not as strong as the effect that could be obtained through other approaches, such as CRISPR/Cas9. However, the latter approach essentially leads to a complete knock-out of the genes of interest and previous work has demonstrated that there are profound differences in relation to *MYB* levels, this being reflected by completely different phenotypes observed in Myb hemizygous, hypomorphic and complete knock-out mouse models [1, 16, 34, 35]. Furthermore, given the importance of Myb in several physiological and pathological processes, a total knock-out would be detrimental for any cell type. A compendium of gene essentialities through CRISPR/Cas9 drop-out screening has been generated for various AML cell lines [36, 37], among which MOLM13 (a sister cell line of MOLM14, derived from the same patient), THP1 and FUJIOKA were also screened. Those data revealed a significant CRISPR score (~2.5) in FUJIOKA cells, but this was roughly half compared to the score determined for THP1 (4.9) and MOLM13 (4.4) (Fig. S5B), suggesting that indeed those cells are less addicted to *MYB* expression and can tolerate better its suppression in comparison to those lines carrying MLL-rearrangements.

The extensive changes in gene expression seen in MLL-driven AML cells in response to *MYB* depletion include the modulation of many genes that are known to be targets of *MYB*, including *PIM1*, *ITGAM*, *GFI1*, *FLT3*, *CD14*, *CCND2*, *S100A9* and *DUSP6*. Most interestingly, MYB depletion is accompanied by a striking increase in the abundance of *MAFB* RNA. A role for MafB in the normal promotion of myeloid differentiation is well established [38] and it is known that Myb can inhibit MafB transactivation potential through direct binding to a SUMOylated form [39]. Taken together, this suggests that MYB normally supports the leukaemia phenotype in MLL-rearranged AML by directly or indirectly limiting MAFB expression, with the additional possibility of suppression of MAFB protein activity, thereby limiting the ability of the cells to differentiate. We sought to assess if MYB would be directly regulating MAFB by being recruited to its promoter, although our ChIP analysis failed to detect any significant enrichment of MYB binding on the MAFB locus (data not shown), possibly owing this to the low sensitivity of the MYB antibody for this kind of approach. However, recent work by Takao and co-workers, employing peptidomimetic blockade of MYB and of its transcriptional coactivator CBP in MLL-r cell lines, demonstrated that MYB inhibition resulted in reduced accessibility to genomic locations enriched with MAF family members consensus motifs and a significant depletion of MYB direct binding to the MAFB locus [31]. These findings support our idea of a direct MYB-mediated repression of MAFB in the context of MLL-rearrangements.

The reduced dependency of the leukaemia phenotype on *MYB* levels in complex karyotype AML cells, represented in our study by FUJIOKA and KG1A cells, is mirrored by a smaller number of changes in gene expression upon *MYB* depletion, including no change in the levels of *MAFB* or known MYB targets such as *GFI1*, *FLT3*, and *PIM1*, which are seen to be affected in the MLL-driven AML cells. The fact that these latter genes are known for their capacity to enforce self-renewal and a myeloid commitment block provides a hint as to why complex karyotype AML might show a minimal response to MYB depletion.

It is worth noting that AML cells characterized by the t(8;21) translocation, represented here by KASUMI1 cells, also responded to *MYB* depletion with a myeloid commitment induction and a reduction of the proliferation capacity, though we failed to detect any *MAFB* expression, suggesting that in these cells *MYB* regulates a distinct programme of gene expression, further highlighting the complexity of aberrant transcriptional programmes brought about by different mutations.

To strengthen the validity of our observations, we extended our bioinformatic analysis to publicly available AML patient gene expression datasets. We stratified patients characterized by either complex karyotype or MLL-rearrangements based on *MYB* or *MAFB* mRNA levels. By comparing low versus high *MYB* expressers, we were able to show transcriptome differences that parallel what we had observed through experimental manipulation of *MYB* in the MOLM14 cells line, with a large number of genes positively and negatively correlated with *MYB* levels, including well-known MYB targets that are normally activated (*BCL2*, *CDK6*, *FLT3* and *GFI1*) or repressed (*BCL6*, *CD14*, *ITGAM* and *DUSP6*) in different cellular contexts. Importantly, this analysis pointed at *MAFB* expression as the most negatively correlated with *MYB* RNA levels, thus further highlighting a significant connection between these two genes.

Given that *MAFB* appeared to be the gene most largely associated with *MYB* expression, we also performed gene expression analysis of patient profiling arrays by stratifying those samples based on the levels of *MAFB* RNA. Interestingly, this produced a large number of both positively and negative correlated genes in MLL-rearranged AML, while very few genes were found to be dependent upon *MAFB* expression in complex karyotype patients. Importantly, this analysis showed that genes whose expression is negatively correlated with *MYB* display a large overlap with genes that are positively correlated with the level of *MAFB* RNA, while conversely, genes positively correlated with *MYB* expression were largely overlapping with those that are negatively correlated with *MAFB*. However, it is worth noting that while the stratification of patients carrying MLL-rearrangements produced similar results using two different cohorts, the same analysis in complex karyotype leukaemias highlighted some considerable differences in terms of numbers of differentially regulated genes in response to either *MYB* or *MAFB* stratification. In fact, while a smaller number of genes was determined from the Haferlach cohorts, a much higher number of genes was observed when stratifying patients from the TARGET-AML cohorts, although no significant association between *MYB* and *MAFB* was determined for those patients. That some MYB target genes (such as *KIT*, *CDK6* and *FLT3*) exhibit a degree of correlation with *MYB* levels amongst the patient datasets probably reflects a wide range of driver mutations and a very heterogeneous genetic landscape amongst the complex karyotype leukaemias. This aspect would certainly require a deeper investigation in a more dedicated study.

In conclusion, this study sheds further light on the dependency of different categories of AML on the level of the MYB transcription factor, demonstrating that, as we had previously described in the case of leukaemias driven by CEBPA mutations [17], there can be vastly different responses to a reduction in *MYB* levels. The finding of a strong inverse correlation between *MYB* and *MAFB* in MLL-driven leukaemia suggests that MAFB could serve as a new useful prognostic biomarker or predictive toward future MYB-targeted therapeutic strategies. Furthermore, MAFB itself could be regarded as an anti-leukaemia factor, which might be amenable to manipulation.

## Reporting summary

Further information on research design is available in the Nature Research Reporting Summary linked to this article.

### Supplementary information

Supplemental information

Reporting Summary

Original Data File

## Data Availability

RNA-Seq data generated in this study are available at the Gene Expression Omnibus (GEO) under series GSE149556. Dataset information can be found in the supplementary digital content.
