# Supplementary material for: Reversal of MYB-dependent suppression of MAFB expression overrides leukaemia phenotype in MLL-rearranged AML
Source: Cell Death Dis. 2023 Nov 23;14(11):763. doi: 10.1038/s41419-023-06276-z (PMC10667525; doi:10.1038/s41419-023-06276-z)
Supplement: Supplementary file 1 — Supplemental information [file 41419_2023_6276_MOESM1_ESM.docx]

**SUPPLEMENTARY INFORMATION**

**Reversal of *MYB*-dependent suppression of *MAFB* expression overrides leukaemia phenotype in MLL-rearranged AML.**

Negri A^1^, Ward C^2^, Bucci A^1^, D’Angelo G^1^, Cauchy P^3^, Radesco A^1^, Ventura A^1^, Walton DS^4^, Clarke M^5^, Mandriani B^6^, Pappagallo SA^1^, Mondelli P^1^, Liao K^7^, Gargano G^8^, Zaccaria GM^9^, Viggiano L^10^, Lasorsa FM^6^, Ahmed A^6^, Di Molfetta D^6^, Fiermonte G^6^, Cives M^11^, Guarini A^1^, Vegliante MC^1^, Ciavarella S^1,12^, Frampton J^5,12,13^, Volpe G^1,12,13^

1. Hematology and Cell Therapy Unit, IRCCS Istituto Tumori “Giovanni Paolo II”, Bari, Italy.
2. Edge Impulse Inc.
3. Max Planck Institute of Immunobiology and Epigenetics, 79108 Freiburg, Germany.
4. Clent Life Sciences, DY84HD, Stourbridge, UK.
5. Institute of Cancer and Genomic Sciences, College of Medical and Dental Sciences, University of Birmingham, B152TT, Birmingham, UK.
6. Department of Bioscience, Biotechnology and Environment, University of Bari, 70125 Bari, Italy.
7. School of Biology and Biological Engineering, South China University of Technology, Guangzhou, 510006.
8. Department of Mathematics, University of Bari Aldo Moro, Bari, Italy.
9. Department of Electrical and Information Engineering, Polytechnic University of Bari, Italy.
10. Department of Biology, University of Bari Aldo Moro, Bari, Italy.
11. Department of Interdisciplinary Medicine, University of Bari, Bari, Italy.
12. Joint Senior authors
13. Corresponding author

**
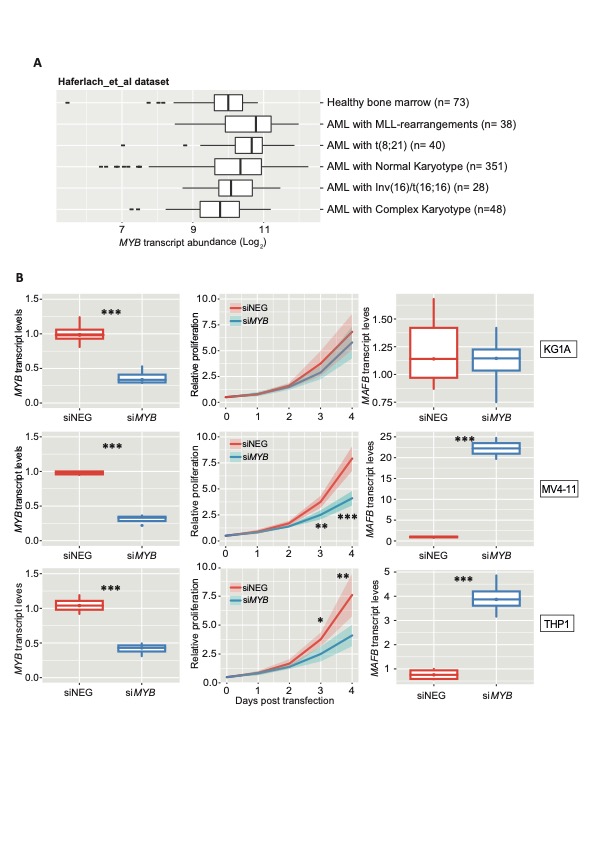
**

**Figure S1. *MYB* depletion in the KG1A, THP1 and MV4-11 cell lines.**

**(A)** Boxplot depicting the abundance of *MAFB* mRNA in subgroups of patients from the Haferlach dataset, characterized by the karyotypic abnormalities indicated in the graph in comparison with healthy bone marrow donors. The statistical significance presented in this plot has been determined by applying a Kruskal-Wallis t-test. (**B)** Box plot depicting the efficiency of *MYB* knock-down in KG1A, MV4-11 and THP1 cell lines by quantitative-PCR determined 24h post transfection in cells transfected with *MYB* siRNA or scrambled negative control. The line plot indicates the cell viability determined by counting cells every 24h for 4 consecutive days. The boxplot on the right shows the abundance of *MAFB* transcript following MYB knock-down. The statistical analysis was performed using Student’s t-test. (***, p<0.001).

**Figure S2. Gene expression consequences of *MYB* depletion**

**(A)** UCSC genome browser screenshots of RNA-seq performed in FUJIOKA, MOLM14 and KASUMI1 cells with either siNEG or si*MYB* treatment at *DUSP6*, *BCL2*, *CD14,* *MYC,* *GFI1*, *CCND2* and *S100A9* loci. Profiles are scaled to 1% GAPDH. **(B)** Euler diagrams showing the numbers and overlap of genes that are either upregulated (left panel) or downregulated (right panel) in response to *MYB* ablation when comparing FUJIOKA (red), MOLM14 (green) and KASUMI1 (blue) cells lines. **(C)** Barplot indicating *MAFB* expression levels in MV4-11 and THP1 cells treated with different MYB inhibitors. **(D)** Barplot indicating the depletion of MYB binding upon treatment with MYB inhibitors at the indicated gene loci.

**Figure S3. MAFB expression levels in human AML patient array.**

Boxplot depicting the abundance of *MYB* transcript in subgroups of patients from the dataset reported by Harferlach et al, characterized by the karyotypic abnormalities indicated in the graph in comparison with healthy bone marrow donors. The statistical significance presented in this plot has been determined by applying a Kruskal-Wallis t-test.

**
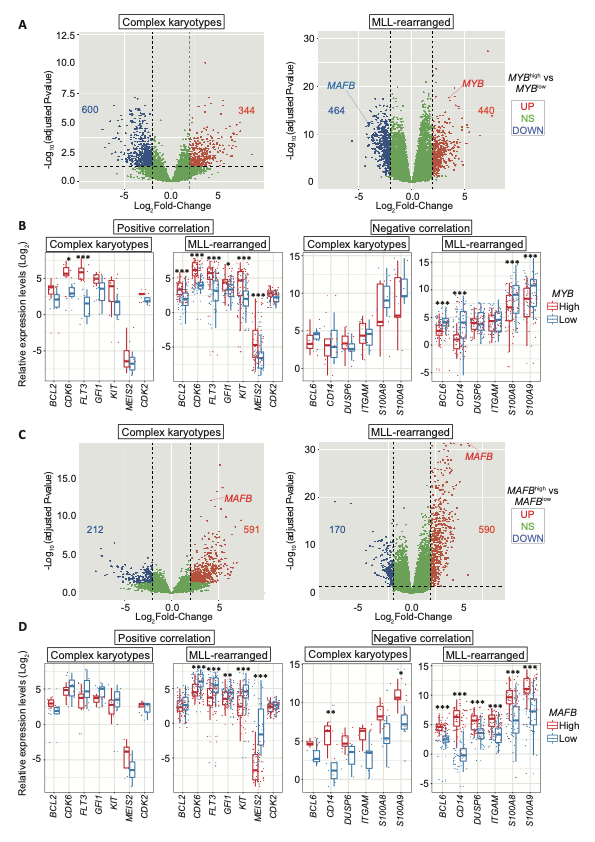
Figure S4. Validation of in silico analysis from the TARGET-AML cohort.**

**(A)** Volcano plot indicating differentially expressed mRNAs with either negative (below -2 Log_2_ FC, indicated as blue dots) or positive (above 2 Log_2_ FC, indicated as red dots) correlation when comparing high versus low *MYB* expressers in each AML subgroup. **(B)** Differential expression of representative *MYB* myeloid target genes (*BCL2, CDK2, CDK6, GFI1, KIT, MEIS2,* *BCL6, CD14, DUSP6, ITGAM, S100A8,* and *S100A9*) comparing low versus high expressers in both complex karyotype and MLL-rearrangements subgroups. Data are presented as overlapping scatter plots in which *MYB*^high^ patients are indicated in red and *MYB*^low^ patients are indicated in blue. **(C)** Volcano plot indicating differentially expressed mRNAs with either negative (below -2 Log_2_ FC, indicated as blue dots) or positive (above 2 Log_2_ FC, indicated as red dots) correlation when comparing high versus low *MAFB* expressers in each AML subgroup. **(D)** Differential expression of representative *MYB* myeloid target genes (*BCL2, CDK2, CDK6, GFI1, KIT, MEIS2,* *BCL6, CD14, DUSP6, ITGAM, S100A8,* and *S100A9*) comparing low versus high expressers in both complex karyotype and MLL-rearrangements subgroups. Data are presented as overlapping scatter plots in which *MAFB*^high^ patients are indicated in red and *MAFB*^low^ patients are indicated in blue. Every plot shows a color-coded boxplot showing a median interquartile range. The statistical analysis shown in each plot indicates p-value adjusted for false discovery rates (***<0.001, **<0.01, *<0.05).

**Figure S5. CRISPR-drop out screening in AML cell lines.**

Bar plot showing the CRISPR score in human AML cell lines treated with *MYB* gRNA from the drop out screen reported by Wang et al.
