# Supplementary figures and images for: Reversal of MYB-dependent suppression of MAFB expression overrides leukaemia phenotype in MLL-rearranged AML
Source: Cell Death Dis. 2023 Nov 23;14(11):763. doi: 10.1038/s41419-023-06276-z (PMC10667525; doi:10.1038/s41419-023-06276-z)

## Slide 1
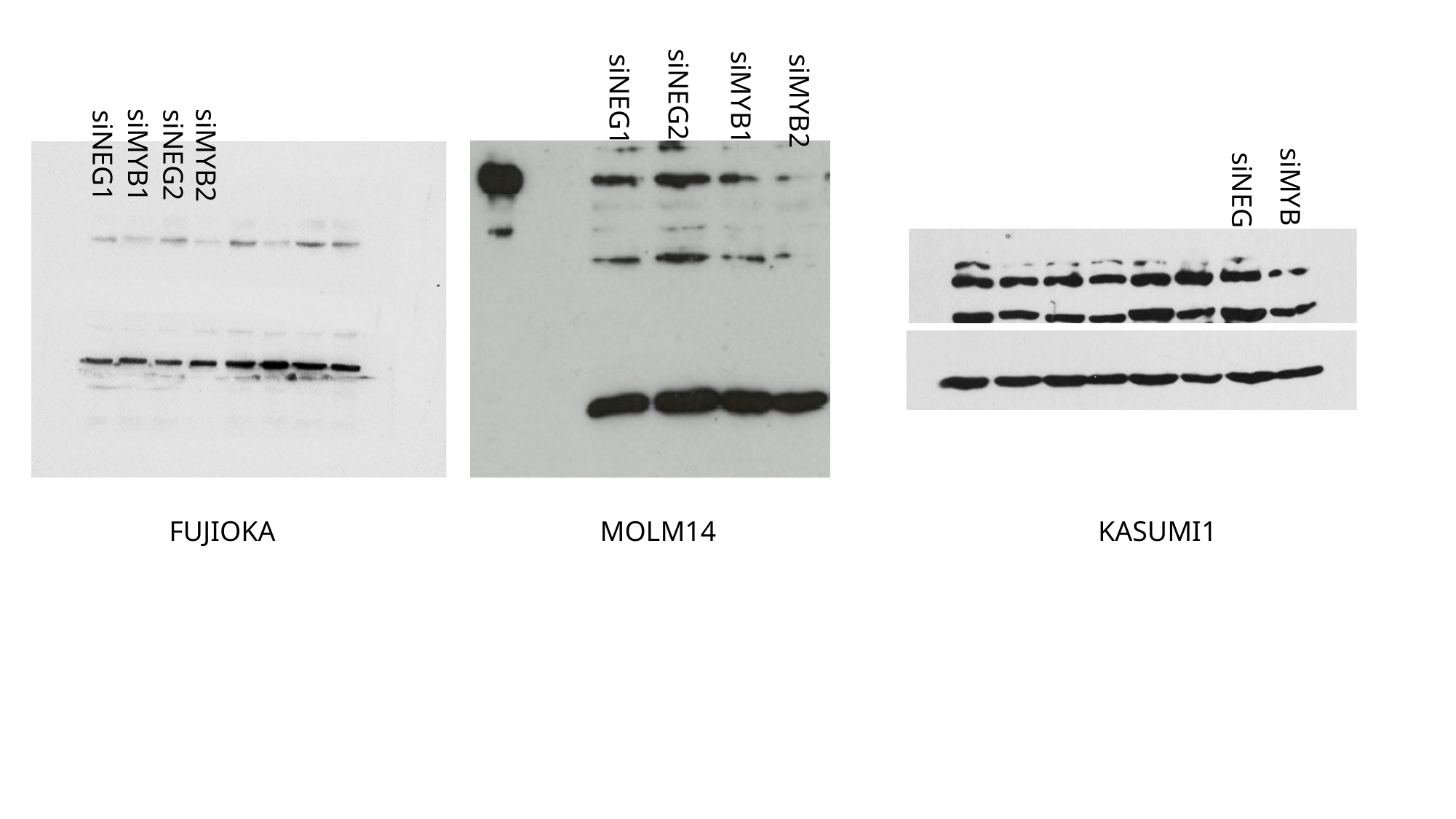

siNEG2
siMYB1
siNEG1
siMYB2
siNEG2
siMYB1
siMYB2
siNEG1
siMYB
siNEG
FUJIOKA
MOLM14
KASUMI1

Supplement: Supplementary file 3 — Original Data File [file 41419_2023_6276_MOESM3_ESM.pptx]
